# Semiautomated pelvic lymph node treatment response evaluation for patients with advanced prostate cancer: based on MET-RADS-P guidelines

**DOI:** 10.1186/s40644-023-00523-4

**Published:** 2023-01-17

**Authors:** Xiang Liu, Zemin Zhu, Kexin Wang, Yaofeng Zhang, Jialun Li, Xiangpeng Wang, Xiaodong Zhang, Xiaoying Wang

**Affiliations:** 1 Department of Radiology, Peking University First Hospital, No.8 Xishiku Street, Xicheng District, Beijing, 100034 China; 2 Department of Hepatobiliary and Pancreatic Surgery, Zhuzhou Central Hospital, Zhuzhou, 412000 China; 3 School of Basic Medical Sciences, Capital Medical University, Beijing, 100069 China; 4 Beijing Smart Tree Medical Technology Co. Ltd, Beijing, 100011 China

**Keywords:** Deep learning, MET-RADS-P criteria, Pelvic lymph nodes, Metastases, DWI

## Abstract

**Background:**

The evaluation of treatment response according to METastasis Reporting and Data System for Prostate Cancer (MET-RADS-P) criteria is an important but time-consuming task for patients with advanced prostate cancer (APC). A deep learning-based algorithm has the potential to assist with this assessment.

**Objective:**

To develop and evaluate a deep learning-based algorithm for semiautomated treatment response assessment of pelvic lymph nodes.

**Methods:**

A total of 162 patients who had undergone at least two scans for follow-up assessment after APC metastasis treatment were enrolled. A previously reported deep learning model was used to perform automated segmentation of pelvic lymph nodes. The performance of the deep learning algorithm was evaluated using the Dice similarity coefficient (DSC) and volumetric similarity (VS). The consistency of the short diameter measurement with the radiologist was evaluated using Bland–Altman plotting. Based on the segmentation of lymph nodes, the treatment response was assessed automatically with a rule-based program according to the MET-RADS-P criteria. Kappa statistics were used to assess the accuracy and consistency of the treatment response assessment by the deep learning model and two radiologists [attending radiologist (R1) and fellow radiologist (R2)].

**Results:**

The mean DSC and VS of the pelvic lymph node segmentation were 0.82 ± 0.09 and 0.88 ± 0.12, respectively. Bland–Altman plotting showed that most of the lymph node measurements were within the upper and lower limits of agreement (LOA). The accuracies of automated segmentation-based assessment were 0.92 (95% CI: 0.85–0.96), 0.91 (95% CI: 0.86–0.95) and 75% (95% CI: 0.46–0.92) for target lesions, nontarget lesions and nonpathological lesions, respectively. The consistency of treatment response assessment based on automated segmentation and manual segmentation was excellent for target lesions [*K* value: 0.92 (0.86–0.98)], good for nontarget lesions [0.82 (0.74–0.90)] and moderate for nonpathological lesions [0.71 (0.50–0.92)].

**Conclusion:**

The deep learning-based semiautomated algorithm showed high accuracy for the treatment response assessment of pelvic lymph nodes and demonstrated comparable performance with radiologists.

## Background

Advanced prostate cancer (APC) is characterized by the recurrence of prostate cancer after definitive treatment or by metastases without prior therapy [1]. Several therapeutic approaches have been approved for patients with APC. Aside from the androgen deprivation and docetaxel treatment, new agents with varying mechanisms of action have shown survival benefits in this population [2, 3]. While the responses of patients with APC to these agents are various and treatment may cause side effects, they may result in the desired outcomes for patients. Therefore, early treatment response assessment for patients with APC allows clinicians to put a timely stop to unbeneficial treatment.

Imagery depicting metastatic state plays a key role in patient management [4, 5]. There is a growing body of research demonstrating how whole-body magnetic resonance imaging can be used to diagnose and evaluate APC tumors and determine the efficacy of treatment [6, 7]. The METastasis Reporting and Data System for Prostate Cancer (MET-RADS-P) guidelines aim to reduce variability in the acquisition, interpretation, and reporting of metastatic cancer by promoting standardization of practices [8]. As recommended by the Prostate Cancer Clinical Trials Working Group (PCWG), MET-RADS-P allows the subclassification of patients based on their metastatic spread pattern (bone, nodal, visceral, or local) [5].

Diffusion-weighted imaging (DWI) has been shown to successfully reflect tumor response and discriminate between future responders and nonresponders, which could be valuable in adapting future management [9]. Manual segmentation and measurement of DWI lesions based on MET-RADS-P require a high level of expertise, are time-consuming, and are subject to operator error [10, 11]. Deep learning technologies have extended this quantitative approach with promising preliminary results in the assessment of tumor response in the liver [12, 13]. In this study, we hypothesized that the deep learning model could also be trained to estimate the treatment response of APC according to MET-RADS-P guidelines. This study aimed to investigate the feasibility of deep learning-based treatment response evaluation of patients with APC, and for proof-of-concept, we focused on the assessment in the pelvic lymph nodes.

## Materials and methods

### Patient enrollment

This study was approved by the local institutional review board, and the requirement for informed consent was waived due to its retrospective design. Two hundred and fifty-nine patients with histologically confirmed prostate cancer who underwent initial/curative treatment of metastases at our institution were included in this study between Jan 2017 and Jan 2022. Pelvic MRI scans were performed before and after at least one course of treatment (baseline and posttreatment).

**Fig. 1:**
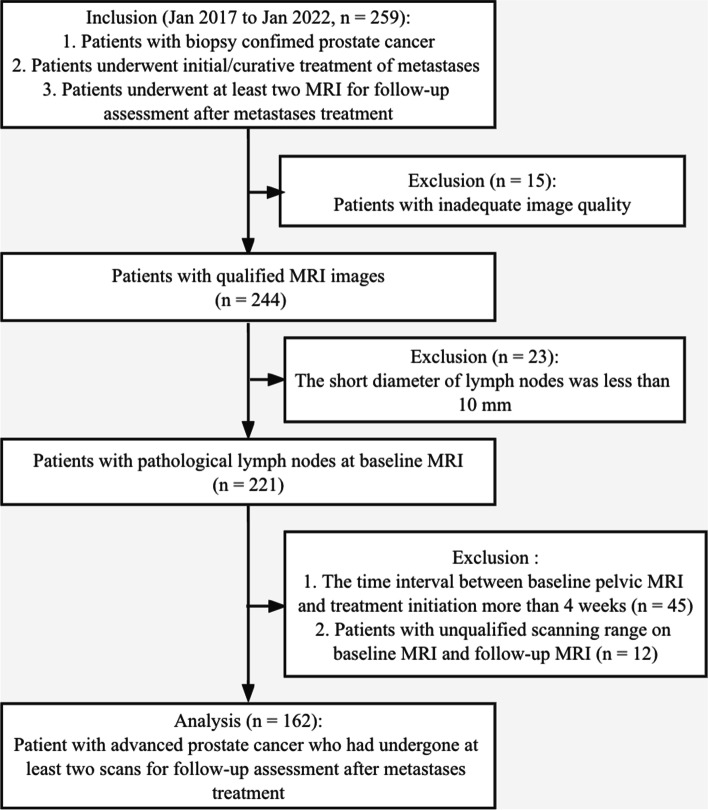
The workflow of patient enrollment

According to the MET-RADS-P criteria, lymph nodes with a short diameter < 10 mm were considered nonpathological; therefore, only patients with lymph nodes ≥ 10 mm at baseline MRI should be included in the protocols. Hence, 23 of the 259 patients with APC were excluded because of the short diameter of all the lesions < 10 mm. In addition, the time interval between baseline pelvic MRI and treatment initiation was suggested to be within 4 weeks; therefore, 45 patients were excluded due to an interval of more than 4 weeks. Twelve patients were excluded because of the unqualified scanning range on baseline and follow-up MRI. Fifteen patients were excluded for inadequate image quality. Finally, 162 patients who had undergone at least two scans for follow-up assessment after APC metastasis treatment were analyzed (Fig. 1). Clinical and radiological features of the enrolled patients were acquired from the electronic information system, including age, prostate-specific antigen (PSA) level, PI-RADS v2.1 scores and TNM staging.

### MRI acquisition

Three 3.0 T scanners were used (Achieva, Philips Healthcare; Discovery MR750, GE Healthcare; Intera, Philips Healthcare) to perform pelvic MRI scans. The pelvic MRI protocol performed in our institution included T2-weighted imaging (T2WI), T1WI, DWI with apparent diffusion coefficient (ADC) maps and dynamic gadolinium-DTPA (Gd-DTPA)-enhanced (DCE) sequences. The detailed scanning parameters of DWI are listed in Table 1.

**Table 1:** The detailed imaging parameters for diffusion-weighted imaging

Parameters	3.0 T Discovery	3.0 T Intera	3.0 T Achieva
B value (s/mm^2^)	0, 800	0, 800	0, 800
Imaging matrix	256 × 256	224 × 224	156 × 180
Echo time (ms)	60	56	54
Repetition time (ms)	4000	6628	3300
Field of view (mm^2^)	450 × 366	662 × 400	512 × 356
Section thickness (mm)	4	4	4
Number of slices	25	20	24

### Pelvic lymph nodes segmentation

A previously trained 3D U-Net segmentation model developed by the same authors in this study based on deep learning was used to automatically segment the visible pelvic lymph nodes on DWI images [14]. The training data used for the model development were different from the data included here. All visible lymph nodes included target lesions (short diameter ≥ 15 mm), nontarget lesions (10 mm ≤ short diameter < 15 mm) and nonpathological lesions (short diameter < 10 mm). Manual corrections of the automatically segmented lymph nodes made by a radiologist expert (with more than 20 years of reading experience) were considered the reference standard for segmentation evaluation.

### Treatment response assessment

Based on the MET-RADS-P criteria, treatment response assessments of lymph nodes were conducted [15], including complete response (CR), partial response (PR), stable disease (SD), and progressive disease (PD).

The radiologists who corrected the lymph nodes manually provided the reference standard for treatment response assessment. An algorithm for semiautomatic response assessment was developed using the MET-RADS-P criteria by automatically calculating the diameters of the lymph nodes first and then assessing the treatment response by a rule-based program. More details about the algorithm development of pelvic lymph nodes were shown in our previous study [14].

**Fig. 2:**
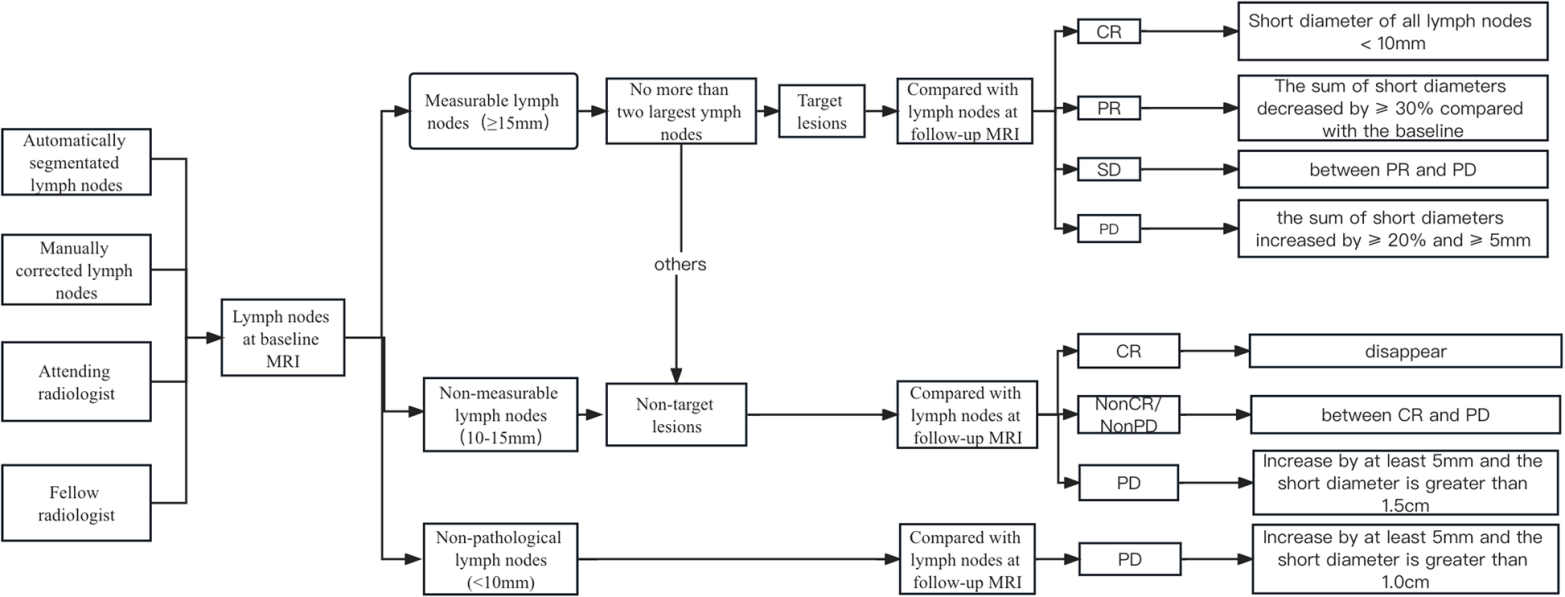
The flowchart of treatment response assessment of lymph nodes

In addition, an attending radiology radiologist (R1) and a fellow radiology radiologist (R2), with 8 and 4 years of pelvic imaging experience, performed the treatment response assessments on all patients by primary review of the MRI images. The two radiologists compared baseline scans before treatment and subsequent scans after treatment for every patient. The definition and evaluation rules are shown in Fig. 2.

### Statistical analysis

The “median (interquartile range)” values are used for the description of continuous variables, and descriptive statistics of the categorical data are presented with “n (%)”. The segmentation results are quantitatively evaluated by the overlap-based metric [Dice similarity coefficient (DSC)] and the volume-based metric [volumetric similarity (VS)] [16]. The independent t-test was applied to determine the difference in the evaluation metrics between the subgroups. We used the Kappa statistic to evaluate the consistency of treatment response. A *P* value less than 0.05 was treated as significant. Statistical analysis was performed with MedCalc (version 14.8; MedCalc Software, Ostend, Belgium).

## Results

### Study population

In this study, 162 eligible APC patients with metastases were included. The baseline characteristics of the enrolled patients are shown in Table 2. The median T-PSA level in this population was 35.39 ng/ml. The PI-RADS scores and T/N/M staging were recorded from the baseline MRI reports, and PI-RADS 5 (74.07%), T4 (30.86%), N1 (56.79%) and M0 (38.89%) accounted for the largest proportion. The Gleason scores were obtained from the pathological report, and Gleason 4 + 5 (37.65%) accounted for the largest percentage.

**Table 2:** Main baseline demographics and clinical characteristics of patients

Characteristics	Value
Age(y)	69 (64, 75)
PSA (ng/ml)	
T-PSA	35.39 (14.2, 70)
F-PSA	4.32 (1.53, 9.00)
F/T-PSA	0.11 (0.07, 0.17)
PI-RADS scores (n%)	
3	7 (4.32%)
4	35 (21.60%)
5	120 (74.07%)
T staging (n%)	
T2	42 (25.93%)
T3a	21 (12.96%)
T3b	49 (30.25%)
T4	50 (30.86%)
N staging (n%)	
X	52 (32.10%)
0	18 (11.11%)
1	92 (56.79%)
M staging (n%)	
X	56 (34.57%)
0	63 (38.89%)
1a	3 (1.85%)
1b	40 (24.69%)
Gleason score (n%)	
3 + 3	10 (6.17%)
3 + 4	10 (6.17%)
3 + 5	6 (3.70%)
4 + 3	24 (14.81%)
4 + 4	25 (15.43%)
4 + 5	61 (37.65%)
5 + 4	23 (14.20%)
5 + 5	3 (1.85%)

All patients had received at least one course of posttreatment MRI examination, 63 patients had two posttreatment examinations, 23 patients had three posttreatment examinations, 8 patients had four posttreatment examinations, 3 patients had five posttreatment examinations, and 1 patient had seven posttreatment examinations. In the baseline pelvic MRI, 112 patients had target lesions, 129 patients had nontarget lesions, and all patients had nonpathological lymph nodes.

### Assessment of automated lymph node segmentation

**Fig. 3:**
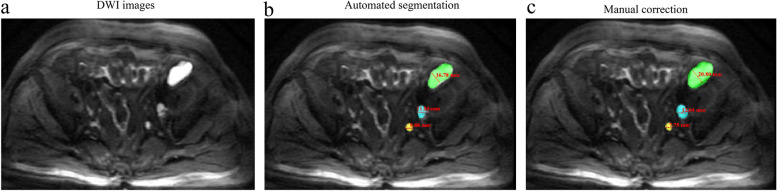
Explementary results of lymph node segmentation and correction. Light green: target lesion; light blue: nontarget lesion; light yellow: nonpathological lesion

One hundred and sixty-two APC patients with 162 baseline pelvic MRI scans and 260 posttreatment MRI scans were used to perform automated lymph node segmentation. As shown in Table 3, the mean DSC and VS are 0.82 ± 0.09 and 0.88 ± 0.12, respectively. In the subgroup analyses, the DSC and VS values of the target lesions and nontarget lesions showed no significant difference (DSC: 0.85 *vs.* 0.82, *P* > 0.05; VS: 0.88 *vs.* 0.86, *P* > 0.05) but were significantly higher than those of nonpathological lesions (all *P* values > 0.05). The subgroups of baseline and posttreatment MRI scans showed no significant difference (all *P* values > 0.05). The explementary segmentation of lymph nodes is shown in Fig. 3.

**Table 3:** Segmentation results of pelvic lymph nodes

Metrics	All	Subgroup analysis				
		Target lesions	Nontarget lesions	Nonpathological lesions	Baseline lesions	Post-treatment lesions
DSC	0.82 ± 0.09	0.85 ± 0.09	0.82 ± 0.09	0.78 ± 0.09	0.81 ± 0.10	0.82 ± 0.09
VS	0.88 ± 0.12	0.88 ± 0.09	0.86 ± 0.08	0.80 ± 0.08	0.87 ± 0.09	0.88 ± 0.08

*DSC* Dice similarity coefficient, *VS* volumetric similarity. The DSC and VS values were used to evaluate the performance of the automated lymph node segmentation by comparison with the manual annotation

### Quantitative measurement of the lymph node segmentation

**Fig. 4:**
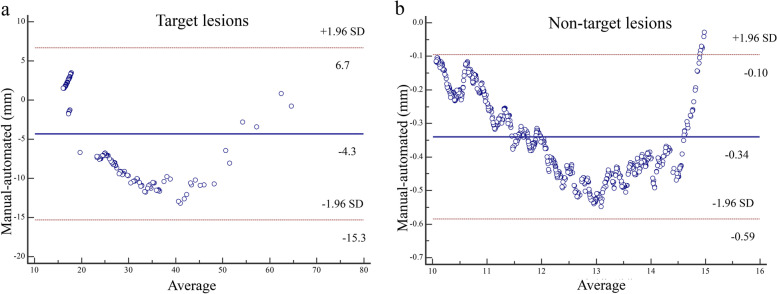
Agreement between the automatically segmented and manually segmented lymph nodes. **a** target lesions; **b** nontarget lesions

The mean short diameters of the automatically segmented and manually segmented target lesions were 23.53 mm (interquartile range, 17.61- 26.55 mm) and 27.94 mm (interquartile range, 15.93—26.77 mm), respectively (*P* = 0.231). The mean short diameters of automatically segmented and manually segmented nontarget lesions were 11.91 mm (interquartile range, 10.85—13.14 mm) and 12.33 mm (interquartile range, 11.07—13.59 mm), respectively (*P* = 0.082). The agreement between the automatically segmented and manually segmented target lesions and nontarget lesions in terms of short diameter is shown in Fig. 4. The Bland–Altman analysis showed good consistency between the automated segmentation and manual segmentation, and most values were within the upper and lower limits of agreement (LOA).

### Accuracy of the treatment response assessment

**Fig. 5:**
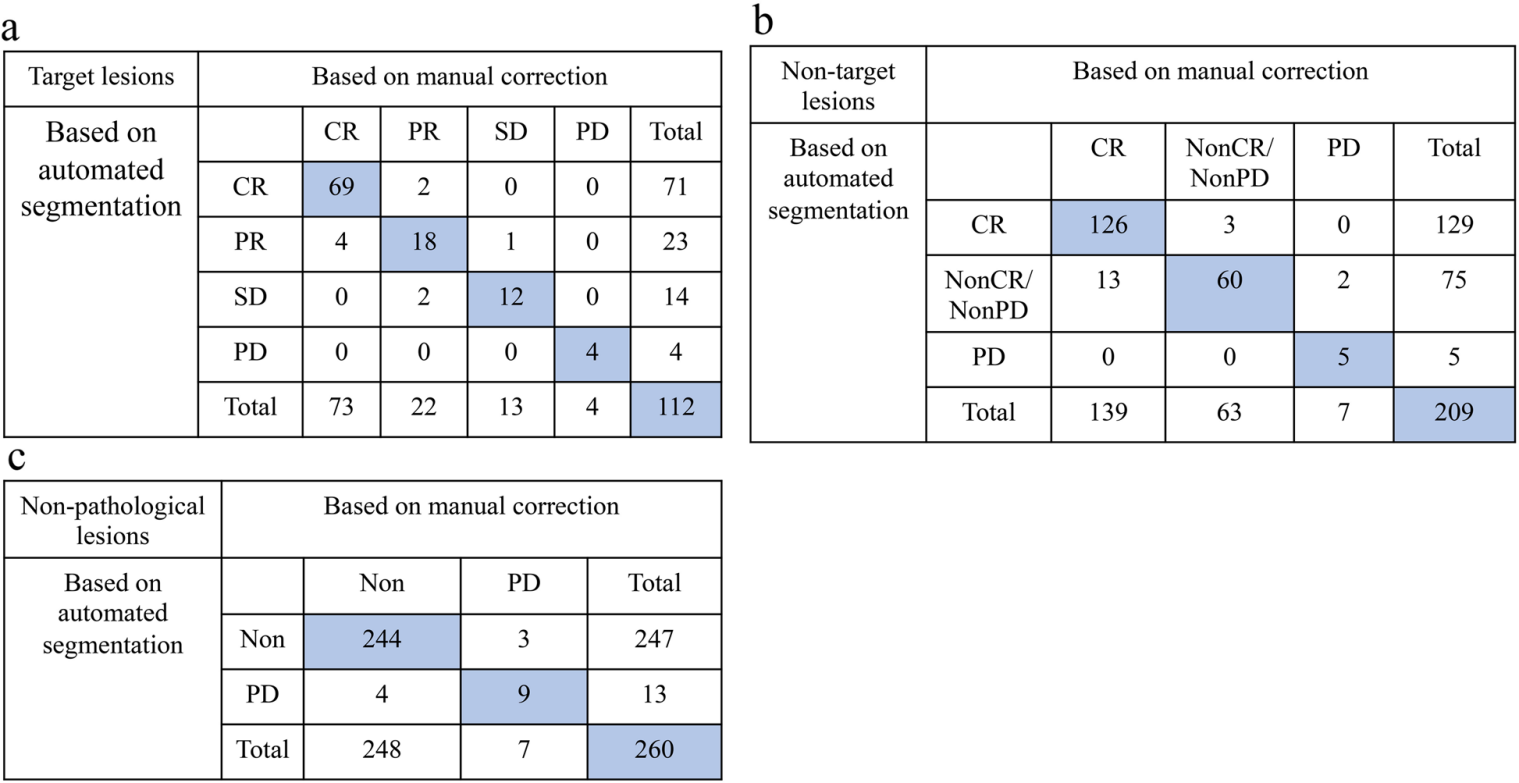
Confusion matrix of treatment response assessment

In this population, 75 APC patients with 112 pairs of pelvic MRI performed the target lesion evaluation; 129 APC patients with 209 pairs of pelvic MRI performed the nontarget lesion evaluation, and 162 APC patients with 260 pairs of pelvic MRI performed the nonpathological lesion evaluation. As shown in Fig. 5, the accuracies of the automated segmentation-based response assessment were 0.92 (95% CI: 0.85–0.96), 0.91 (95% CI: 0.86–0.95) and 75% (95% CI: 0.46–0.92) for target lesions, nontarget lesions and nonpathological lesions, respectively.

### Consistency of the treatment response assessment

As shown in Table 4, the agreement of treatment response assessment based on automated segmentation and manual correction was excellent for target lesions [*K* value: 0.92 (0.86–0.98)], good for nontarget lesions [0.82 (0.74–0.90)] and moderate for nonpathological lesions [0.71 (0.50–0.92)], which were approximately equal to the agreement between R1 and manual correction [0.89, 0.81 and 0.68 for target lesions, nontarget lesions and nonpathological lesions, respectively] but slightly higher than the agreement between R2 and the reference standard [0.86, 0.82 and 0.60 for target lesions, nontarget lesions and nonpathological lesions, respectively].

**Table 4:** The consistency of treatment response assessment

Comparison	Target lesions	Nontarget lesions	Nonpathological lesions
Automated segmentation *vs.* Manual correction	0.92 (0.86–0.98)	0.82 (0.74–0.90)	0.71 (0.50–0.92)
R1 *vs.* Automated segmentation	0.89 (0.81–0.96)	0.81 (0.73–0.89)	0.68 (0.47–0.88)
R2 *vs.* Automated segmentation	0.86 (0.79–0.94)	0.78 (0.69–0.87)	0.60 (0.37–0.82)
R1 *vs.* R2	0.90 (0.85–0.97)	0.95 (0.90–0.99)	0.84 (0.68–0.99)
R1 *vs.* Manual correction	0.96 (0.93–1.00)	0.99 (0.97–1.00)	0.96 (0.88–1.00)
R2 *vs.* Manual correction	0.94 (0.89–0.99)	0.96 (0.92–0.99)	0.88 (0.73–1.00)

*R1* an attending radiologist with 8 years of reading experience, *R2* a fellow radiologist with 4 years of reading experience

## Discussion

MET-RADS-P is a guideline for the treatment response evaluation of systemic metastases of patients with APC, which involves the evaluation of primary focus, bone metastases, lymph node metastases and organ metastases. In this study, we established a semiautomatic pelvic lymph node treatment response evaluation process for patients with APC through lymph node segmentation based on deep learning. Our results showed that the accuracies of automated segmentation-based response assessment were high for all the target lesions, nontarget lesions and nonpathological lesions according to MET-RADS-P criteria and achieved good consistency with the attending radiologist and fellow radiologist.

Based on the morphology and signal characteristics of all acquired images, the MET-RADS-P system mapped unequivocal diseases to 14 predefined body regions [8, 15]. Analysis of lymph node metastases in the pelvis is crucial for clinical practice and drug studies in patients with APC, which is the most common metastatic site [17]. A lymph node's size is highly correlated with survival time, a measurement that radiologists and clinicians perform to monitor disease progression or assess therapeutic options, due to the fact that many malignancies can enlarge lymph nodes [18]. According to the Response Evaluation Criteria in Solid Tumors 1.1 (RECIST 1.1) Guidelines, lymph nodes with a short-axis diameter of at least 10 mm are considered to be enlarged lymph nodes and are clinically significant [19]. The size standard of pathological lymph nodes defined by MET-RADS-P based on MRI was similar to RECIST 1.1, while MET-RADS-P provides a more complete assessment of nodal metastases response including the nontarget nodes and nonpathologic nodes, which was usually qualitatively assessed by RECIST 1.1 criteria.

According to the MET-RADS-P criteria, the core whole body MRI protocol designed for bone and lymph node metastasis detection included T1WI (GRE Dixon technique) and axial DWI [8]. DWI is a well-recognized and used sequence for pelvic lymph node imaging, that is able to offer qualitative and quantitative assessments for disease characterizations [14, 20]. Therefore, in this study, we performed the treatment response assessment only on DWI images.

In this study, the established semiautomatic pelvic lymph node treatment response evaluation process according to MET-RADS-P criteria included two parts. First, a previously established pelvic lymph node segmentation model was used to perform the automatic segmentation of lymph nodes. The model achieved good segmentation performance here, which is similar to the segmentation results reported in previous literature (the DSC and VS values for all visible lymph nodes were 0.76 ± 0.15 and 0.82 ± 0.14, respectively) [14], especially the target lesions, further highlighting its potential usefulness.

Second, based on the quantitative measurements obtained from the automated segmentation, we can directly evaluate the treatment response according to MET-RADS-P criteria, which can be more practical in clinical settings. A clinical radiology report provides a qualitative narrative, but does not provide standardized, quantitative information about the patient's progress or response to treatment [21]. Natural language processing and deep learning models have been employed in previous studies to estimate responses from clinical text [22, 23]. These approaches can be feasible for quantitative assessment related to MET-RADS-P criteria but can be indirect.

Our proposed semiautomated algorithm achieved high Kappa values in terms of treatment response assessment with attending and fellow radiologists when measuring the same set of target and nontarget lesions. The consistency of nonpathological lesions was lower, which may be due to the relatively poor segmentation performance. Tang et al. [24] proposed a deep learning-based method for semiautomated RECISTS measurement and assessed using a mean difference between the deep learning algorithm and manual measurement in the unit of pixels. Scores using pixel difference, however, may not be reliable, as scores are largely determined by data composition. In this study, we used Bland–Altman plotting based on percent measurement difference to address the issue as suggested by Woo et al. [25]. As demonstrated, the Bland–Altman analysis indicated good consistency between the automated segmentation and manual segmentation, and most values were within the upper and lower LOA.

There are some limitations that need to be addressed. First, in this study, the deep learning-based treatment response assessment was only focused on the pelvic lymph node, and other regions of the body according to the MET-RADS-P guideline need to be investigated in the future. Second, we acknowledge that there remain opportunities for further model refinement, including the achievement of lymph node registration between baseline and posttreatment images, thus realizing fully automated lymph node treatment response evaluation. Finally, our results demonstrated that the semiautomated treatment response assessment can be achieved on the DWI sequence, but the values of other sequences (e.g. T1WI, DCE or T2WI) on response assessment also need to be investigated in further studies.

## Conclusion

In conclusion, we have developed a semiautomated deep learning-based model to estimate response assessments of pelvic lymph nodes in patients with APC. The accuracy of response assessments based on the automatically segmented lymph nodes showed close similarity to the manually segmented lymph nodes and yielded output comparable to the radiologists. These initial results provide a promising way to achieve a fully automated treatment response assessment algorithm according to MET-RADS-P criteria.

## Data Availability

The datasets used and/or analyzed during the current study are available from the corresponding author on reasonable request.

## Abbreviations

ADC: Apparent diffusion coefficient
APC: Advanced prostate cancer
CR: Complete response
DCE: Dynamic contrast-enhanced
DSC: Dice similarity coefficient
DWI: Diffusion-weighted imaging
LOA: Limits of agreement
MET-RADS-P: METastasis Reporting and Data System for Prostate Cancer
PD: Progressive disease
PR: Partial response
PCWG: Prostate Cancer Clinical Trials Working Group
PSA: Prostate-specific antigen
SD: Stable disease
T2WI: T2-weighted imaging
VS: Volumetric similarity
